# Neural oscillation in low-rank SNNs: bridging network dynamics and cognitive function

**DOI:** 10.3389/fncom.2025.1598138

**Published:** 2025-06-04

**Authors:** Bin Li, Tianyi Zheng, Reo Otsuki, Masato Sugino, Kenta Shimba, Kiyoshi Kotani

**Affiliations:** ^1^Graduate School of Frontier Sciences, The University of Tokyo, Chiba, Japan; ^2^Graduate School of Engineering, The University of Tokyo, Tokyo, Japan

**Keywords:** spiking neural networks, recurrent neural networks, gamma oscillation, low-rank, non-linear dynamics, bifurcation analysis, neural computation, cognitive function

## Abstract

Neural oscillation, particularly gamma oscillation, are fundamental to cognitive processes such as attention, perception, and decision-making. Experimental studies have shown that the phase of gamma oscillation modulates neuronal response selectivity, suggesting a direct link between oscillatory dynamics and cognition. However, there remains a lack of computational models that can systematically simulate and investigate this effect. To address this, we construct a low-rank spiking neural network (low-rank SNN) based on the voltage-dependent theta model to explore how structured connectivity shapes oscillatory dynamics and cognitive function. Using macroscopic model analysis, we identify different network states, ranging from stationary firing to gamma oscillation. Our model successfully reproduces phase-dependent response modulation in a Go-Nogo task, consistent with *in vivo* findings, providing an explanation for how neural oscillation influences task performance. Besides phase dependency, our findings suggest that gamma oscillation can enhance and prolong signal response. Compared to prior studies that applied low-rank connectivity to SNNs but remained limited to stationary or weak oscillatory regimes, our work extends to population-level synchronous activity while maintaining biological plausibility under Dale's principle. Our study offers a theoretical framework for understanding how neural oscillations emerge in structured spiking networks and provides a foundation for future experimental and computational investigations into oscillatory modulation of cognition.

## 1 Introduction

A central goal in computational neuroscience is to understand how networks of interconnected neurons give rise to complex cognitive functions. Recent studies suggest that the brain performs computations through low-dimensional population dynamics embedded within high-dimensional neural activity (Ko et al., 2011; Vyas et al., 2020; Sussillo et al., 2015). In this context, low-rank recurrent neural networks (low-rank RNNs) have emerged as a powerful modeling framework. The work of Mastrogiuseppe and Ostojic demonstrated that adding low-rank structured connectivity to randomly connected RNNs can constrain the network's activity to a low-dimensional subspace, making it both interpretable and analytically tractable (Mastrogiuseppe and Ostojic, 2018). These networks can be designed to perform specific tasks, effectively linking connectivity structure, network dynamics, and function. Moreover, trained RNNs have been shown to be well approximated by low-rank models without loss of performance (Schuessler et al., 2020), highlighting their utility in modeling cognitive computation.

At the same time, neural oscillations–especially gamma oscillations (30-100 Hz)–is widely observed in cortical circuits and play critical roles in cognitive processes such as attention, working memory, and decision-making (Rodriguez et al., 1999; Alekseichuk et al., 2016). These oscillations arise from the synchronized activity of neuronal populations and are thought to contribute to information routing and integration. Disruptions in gamma rhythms have been linked to neurological disorders such as epilepsy (Hughes, 2008) and schizophrenia (Cho et al., 2006). Notably, electrophysiological recordings have shown that neuronal selectivity in the visual cortex can vary with the phase of gamma oscillations (Womelsdorf et al., 2012), suggesting that the timing of oscillatory activity may modulate sensory processing and behavioral performance.

Despite growing interest in low-rank network models and their ability to capture low-dimensional dynamics, their application to spiking neural networks (SNNs)–which capture biologically realistic temporal dynamics–remains relatively limited. In particular, the potential of low-rank SNNs to generate oscillatory activity and explain phase-dependent modulation of cognitive tasks has not been fully explored. Some previous models have extended low-rank connectivity to SNNs, but often without adhering to biological constraints such as Dale's principle, which requires that excitatory and inhibitory neurons exert only positive or negative synaptic effects, respectively (Cimeša et al., 2023). Additionally, prior work has rarely examined population-level oscillatory dynamics or the role of oscillation phase in shaping network responses.

In this study, we construct low-rank spiking neural networks using the voltage-dependent theta neuron model, while strictly enforcing Dale's principle. We first analyze the macroscopic dynamics of the model using bifurcation analysis, revealing how the probability of recurrent connections and the strength of external input jointly determine the frequency of emergent oscillations. We then focus on networks with rank-1 structured connectivity and examine their behavior in a Go-Nogo task–a well-established cognitive paradigm testing the ability to respond selectively to specific stimuli. Our simulations show that the phase of ongoing gamma oscillation strongly modulates the network's response to external inputs, in line with experimental findings. These results demonstrate how low-rank structure can work under the gamma oscillation and gamma oscillation influence the functional output from spiking networks, providing a computational framework for studying the link between neural synchrony and cognitive function.

## 2 Materials and methods

### 2.1 Low-rank networks and Go-Nogo task

Previous research (Mastrogiuseppe and Ostojic, 2018) has studied how low-rank connectivity in rate-based recurrent neural networks (RNNs) contributes to their computational capabilities. The dynamics of such networks are described by:


(1) $$\begin{array}{l} \frac{dx_{i}\left(t\right)}{dt}=-x_{i}\left(t\right)+\overset{N}{\underset{j=1}{\sum }}J_{ij}\phi \left(x_{j}\left(t\right)\right)+I_{i} \end{array}$$

where *x*_*i*_ is the internal state of neuron *i*, interpreted as the input current; *N* is the total number of neurons; **J** ∈ ℝ^*N*×*N*^ is the connectivity matrix; *J*_*ij*_ represents the synaptic weight from neuron *j* to *i*; ϕ(·) = tanh(·) is the activation function; and *I*_*i*_ denotes external input to neuron *i*.

The connectivity matrix **J** is composed of two parts: a random matrix **χ** and a low-rank structured matrix **P**:


(2) $$\begin{array}{l} \text{J}=\lambda \chi +\text{P} \end{array}$$

(3) $$\begin{array}{l} \text{P}=m\cdot n^{T} \end{array}$$

Here, λ = 1 controls the strength of the random component, while ***m***, ***n*** ∈ ℝ^*N*^ are vectors sampled from a Gaussian distribution, referred to as right and left connectivity vectors, respectively ($m_{i}\sim N\left(0,\sigma^{2}/N\right),\;n_{i}\sim N\left(0,\sigma^{2}/N\right)$).

We use the Go-Nogo task to evaluate the network's ability to distinguish between stimuli. To investigate the network's immediate response, we adopted an immediate-response version of the Go-Nogo task, which differs from the classical paradigm. In this version, the network is required to generate an output immediately upon receiving the task signal. The “Go” input is aligned with ***n***, i.e., **I**_*go*_ = ***n***, while the “Nogo” input is orthogonal to it, **I**_*nogo*_ ⊥ ***n*** (${\text{I}}_{nogo,i}\sim N\left(0,\sigma^{2}/N\right)$). The readout vector is set to **W**_*out*_ = ***m***, so the network output reflects the projection of network activity along **W**_*out*_. This structure ensures a strong response to “Go” inputs and a weak response to “Nogo” inputs (Mastrogiuseppe and Ostojic, 2018).

### 2.2 Voltage-dependent theta model

To simulate biologically realistic oscillatory activity, we adopted the voltage-dependent theta model derived from the quadratic integrate-and-fire (QIF) neuron model (Kotani et al., 2014). This model transforms the membrane voltage into a phase variable, enabling the description of periodic firing while retaining biophysical plausibility.

The membrane voltage $V_{X}^{\left(i\right)}$ of neuron *i* in population *X* (either excitatory or inhibitory) evolves according to:


(4) $$\begin{array}{l} C\frac{dV_{X}^{\left(i\right)}\left(t\right)}{dt}=-g_{LX}\frac{\left(V_{X}^{\left(i\right)}\left(t\right)-V_{R}\right)\left(V_{X}^{\left(i\right)}\left(t\right)-V_{T}\right)}{V_{T}-V_{R}}+\left(I_{X}+I_{task}\right) \end{array}$$

(5) $$\begin{array}{l} -\sum_{j=1}^{N_{E}}g_{E\left(j\right)}^{X\left(i\right)}\left[V_{X}^{\left(i\right)}\left(t\right)-V_{E}\right] \end{array}$$

(6) $$\begin{array}{l} -\sum_{j=1}^{N_{I}}g_{I\left(j\right)}^{X\left(i\right)}\left[V_{X}^{\left(i\right)}\left(t\right)-V_{I}\right] \end{array}$$

Here, *C* = 1μF/cm^2^ is the membrane capacitance; *V*_*R*_ = −62 mV and *V*_*T*_ = −55 mV are the resting and threshold potentials, respectively; *g*_*LX*_ is the leak conductance for population *X*; and *I*_*X*_ is the baseline input current sampled from a Cauchy-Lorentz distribution $r\left(I\right)=\frac{1}{\pi }\frac{\Delta }{{\left(I-\eta \right)}^{2}+\Delta^{2}}$, where η and Δ represent the center and width of the distribution. Task-related input is denoted as *I*_*task*_ (**I**_*go*_ or **I**_*nogo*_ in Go-Nogo task), while $g_{E\left(j\right)}^{X\left(i\right)}$ and $g_{I\left(j\right)}^{X\left(i\right)}$ denote the conductance from excitatory and inhibitory presynaptic neurons, respectively. *V*_*E*_ = 0 mV and *V*_*I*_ = −70 mV are the excitatory and inhibitory reversal potentials.

We transform the membrane voltage to a phase variable via:


(7) $$\begin{array}{l} V^{\left(i\right)}=\frac{V_{R}+V_{T}}{2}+\frac{V_{T}-V_{R}}{2}\tan\left(\frac{\theta_{X}^{\left(i\right)}}{2}\right) \end{array}$$

With the transformation in Equation 7, we convert the membrane potential *V* into a phase variable θ. As the properties of the tangent function, when θ crosses π, the membrane potential V undergoes a jump from positive infinity to negative infinity. This allows the continuous evolution of θ to produce a discontinuous jump in V. For numerical implementation, we employ event detection: whenever θ≥π, we record a spike and reset the phase according to θ←θ−2π, which is mathematically equivalent to the spike/reset discontinuity in the QIF model (Kotani et al., 2014; Kopell and Ermentrout, 1986).

Using this transformation and the parameter definitions:


(8) $$\begin{array}{l} c_{0}=\frac{2}{V_{T}-V_{R}}, \end{array}$$

(9) $$\begin{array}{l} c_{1}=\frac{2V_{E}-V_{T}-V_{R}}{V_{T}-V_{R}}, \end{array}$$

(10) $$\begin{array}{l} c_{2}=\frac{2V_{I}-V_{T}-V_{R}}{V_{T}-V_{R}}, \end{array}$$

(11) $$\begin{array}{l} g_{XE}^{\left(i\right)}=\sum_{j=1}^{N_{E}}g_{E\left(j\right)}^{X\left(i\right)}, \end{array}$$

(12) $$\begin{array}{l} g_{XI}^{\left(i\right)}=\sum_{j=1}^{N_{I}}g_{I\left(j\right)}^{X\left(i\right)} \end{array}$$

we derive the voltage-dependent theta model:


(13) $$\begin{array}{l} C\frac{d\theta_{X}^{\left(i\right)}}{dt}=-g_{LX}\cos\theta_{X}^{\left(i\right)}+c_{0}\left(1+\cos\theta_{X}^{\left(i\right)}\right)\left(I_{X}+I_{task}\right)\\ +g_{XE}^{\left(i\right)}\left[c_{1}\left(1+\cos\theta_{X}^{\left(i\right)}\right)-\sin\theta_{X}^{\left(i\right)}\right]\\ +g_{XI}^{\left(i\right)}\left[c_{2}\left(1+\cos\theta_{X}^{\left(i\right)}\right)-\sin\theta_{X}^{\left(i\right)}\right] \end{array}$$

Synaptic conductances evolve via a first-order exponential filter:


(14) $$\begin{array}{l} \frac{dg_{XY}^{\left(i\right)}}{dt}=-\frac{1}{\tau_{d}}g_{XY}^{\left(i\right)}\left(t\right)+\frac{1}{\tau_{d}}g_{XY}^{peak}\overset{N_{Y}}{\underset{j=1}{\sum }}J_{ij}\delta \left(t-t^{j}\right) \end{array}$$

where τ_*d*_ is the synaptic decay time constant, $g_{XY}^{peak}$ is the peak conductance from population *Y* to *X*, and *t*^*j*^ is the spike time of neuron *j*. We determine whether a neuron fires by checking whether its phase variable θ crosses π. Equation 14 is corresponding to Equation 1 in the construction of our low-rank SNN. All parameter values used in the model are summarized in Table 1.

**Table 1:** Parameters in voltage-dependent theta model.

**Symbol**	**Property**	**Default value**
*N*_*X*_	Number of neurons	*N*_*I*_ = 200 *N*_*E*_ = 800
*C*	Membrane capacitance (μF/cm^2^)	1
τ_*d*_	Synaptic decay time (ms)	2 (excitatory synapse) 5 (inhibitory synapse)
*g*_*LX*_	Leakage conductance (mS/cm^2^)	*g*_*LI*_ = *g*_*LE*_ = 0.1
*V*_*R*_	Resting potential (mV)	−62
*V*_*T*_	Threshold potential (mV)	−55
*V*_*X*_	Synaptic reversal potential (mV)	*V*_*I*_ = −70 *V*_*E*_ = 0
*I*_*X*_	Background input to population *X* (μA/cm^2^)	*I*_*X*_~Cauchy(η, Δ) η_*I*_ = η_*E*_ = η = 2 Δ_*E,I*_ = 0.04
*I*_*task*_	Task-related input (μA/cm^2^)	**I**_*go*_ or **I**_*nogo*_
$g_{XY}^{peak}$	Peak synaptic conductance from Y to X (mS/cm^2^)	$g_{EE}^{peak}=0.00407$ $g_{EI}^{peak}=0.02672$ $g_{IE}^{peak}=0.003276$ $g_{II}^{peak}=0.02138$

### 2.3 Bifurcation analysis

To guide parameter selection for oscillatory regimes, we conduct bifurcation analysis based on a macroscopic version of the theta model (Zheng et al., 2021). Unlike microscopic dynamics where conductances vary by neuron, the macroscopic model assumes uniform conductance *g*_*XY*_(*t*) for each population pair:


(15) $$\begin{array}{l} \frac{dg_{XY}\left(t\right)}{dt}=-\frac{1}{\tau_{Y}}g_{XY}\left(t\right)+g_{XY}^{\text{peak}}p_{XY}N_{Y}A_{Y}\left(t\right) \end{array}$$

where *p*_*XY*_ is the connection probability and *A*_*Y*_(*t*) is the firing rate of population *Y*.

The firing rate *A*_*X*_(*t*) is derived from the first-order moment *z*_*X*_(*t*) using:


(16) $$\begin{array}{l} A_{X}\left(t\right)=\frac{g_{L}^{X}}{2\pi C}\left[1+2\text{Re}\left(\frac{-z_{X}\left(t\right)}{1+z_{X}\left(t\right)}\right)\right] \end{array}$$

The temporal evolution of *z*_*X*_(*t*) follows:


(17) $$\begin{array}{l} \frac{d}{dt}z_{X}\left(t\right)=i\left[f_{X}z_{X}{\left(t\right)}^{2}+h_{X}z_{X}\left(t\right)+f_{X}^{*}\right] \end{array}$$

with complex coefficients *f*_*X*_, *h*_*X*_ defined as:


(18) $$\begin{array}{l} f_{X}=\frac{1}{2C}\left(-g_{L}+c_{1}\left(\eta +i\Delta \right)+\underset{Y}{\sum }c_{2\left(Y\right)}g_{XY}\left(t\right)\right)\\ +i\frac{1}{2C}\underset{Y}{\sum }g_{XY}\left(t\right) \end{array}$$

(19) $$\begin{array}{l} h_{X}=\frac{1}{C}\left(c_{1}\left(\eta +i\Delta \right)+\underset{Y}{\sum }c_{2\left(Y\right)}g_{XY}\left(t\right)\right) \end{array}$$

By tracking when a Hopf bifurcation occurs, we can determine the onset of population-level oscillations. All bifurcation diagrams are generated using XPPAUT (Ermentrout, 2002). Simulation code is available at: https://github.com/LiBinUtokyo/LowRank_ModifiedTheta_SNN.

### 2.4 Construction of low-rank SNNs

Based on the model introduced in Sections 2.1, 2.2, we propose our construction of biologically plausible low-rank spiking neural networks (SNNs) that operate in the gamma oscillation regime and follow Dale's principle.

In our model, low-rank structure is applied exclusively to the excitatory-to-excitatory (E → E) connections. This choice is motivated by biological evidence that excitatory neurons serve as the principal long-range projection cells in cortical circuits, while inhibitory neurons primarily play a modulatory role. Accordingly, task-related inputs are applied to the excitatory population, and the network output is read from the excitatory-to-excitatory synaptic currents.

The connectivity matrix **J** governing the interaction strengths among neurons is constrained to 0 ≤ *J*_*ij*_ ≤ 1 and is constructed through Equation 2. Since excitatory and inhibitory effects are embedded in the synaptic dynamics (Equations 13, 14), there is no need to encode sign constraints directly into **J**. Thus, Dale's principle is naturally preserved.

As to the construction of the connectivity matrix **J**. We first construct a rank-one matrix **P** by sampling *N*_*E*_-dimensional vectors ***m*** and ***n*** from a Gaussian distribution (μ = 0, σ = 0.2), and forming **P** via Equation 3. To ensure the resulting SNN operates in the gamma range (around 40 Hz), we first estimate average connection strengths between populations using bifurcation analysis. These estimates are then used to calibrate the random matrix **χ** such that the mean of the final connectivity matrix **J** = **χ**+**P** (Equation 2) satisfies the required average connection strengths for specific population activity pattern according to bifurcation analysis. Random weights in **χ** are sampled from a Gamma distribution with standard deviation 0.1. To ensure that *J*_*ij*_ ∈ [0, 1], we truncate values below 0 to 0 and above 1 to 1. For large *N*, this truncation does not significantly affect the mean values.

The network output *Z*(*t*) is defined as the projection of the total excitatory synaptic current onto the readout vector **W**_*out*_:


(20) $$\begin{array}{l} Z\left(t\right)=\overset{N_{E}}{\underset{i=1}{\sum }}W_{out}^{i}\cdot \tanh\left(I_{EE}^{syn,i}\left(t\right)\right) \end{array}$$

(21) $$\begin{array}{l} I_{EE}^{syn,i}\left(t\right)=-g_{EE}^{i}\left(t\right)\left(V^{i}\left(t\right)-V_{E}\right) \end{array}$$

where $I_{EE}^{syn,i}\left(\mu {\text{A/cm}}^{2}\right)$ denotes the total synaptic current from the excitatory population to excitatory neuron *i*, $g_{EE}^{i}\left({\text{mS/cm}}^{2}\right)$ represents the total synaptic conductance from the excitatory population to excitatory neuron *i*, and *V*^*i*^(*t*) is the membrane potential of excitatory neuron *i*. Additionally, we define the output energy as: *E*(*t*) = *Z*(*t*)^2^, where *Z*(*t*) is the projection of the excitatory synaptic current vector onto the readout direction. Using this construction, we analyze how the oscillatory regime modulates cognitive task performance in a structured spiking network.

## 3 Results

### 3.1 Predicting population activity patterns via bifurcation analysis of the macroscopic model

**Figure 1:**
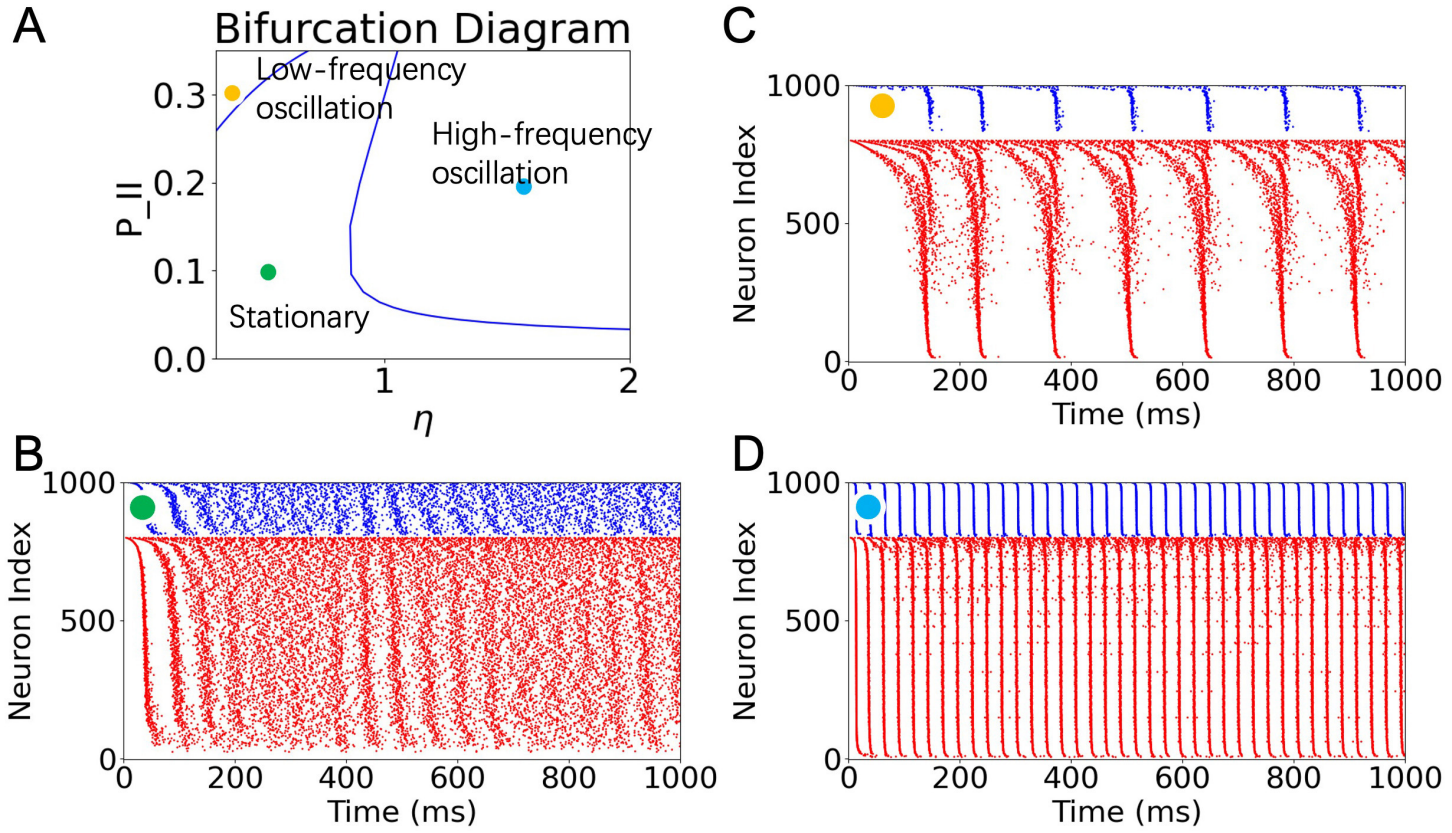
Predictions from bifurcation analysis and validation with SNN simulations. **(A)** Two-parameter bifurcation diagram showing population dynamics of inhibitory neurons as a function of background input η and inhibitory recurrent connectivity *P*_*II*_. The diagram reveals three distinct regions: stationary (green), low-frequency oscillation (yellow), and high-frequency oscillation (blue). **(B–D)** Raster plots of network activity in a low-rank SNN under the three sampled conditions indicated in **(A)**: **(B)** Stationary state for (η, *P*_*II*_) = (0.4, 0.1). **(C)** Low-frequency oscillation for (η, *P*_*II*_) = (0.1, 0.3). **(D)** High-frequency oscillation for (η, *P*_*II*_) = (1.5, 0.2). Each simulation involves 800 excitatory (red) and 200 inhibitory (blue) neurons. See Supplementary Figure S2 for the validation of computational capability for stationary and low-frequency oscillation states.

Exhaustively searching the parameter space of a neuronal network model to identify activity regimes can be computationally intensive and inefficient. Alternatively, macroscopic approximations provide valuable insight into how network dynamics are shaped by key parameters. In this study, we performed a bifurcation analysis of the macroscopic model to investigate how the firing patterns of the inhibitory neuronal population depend on two parameters: the background input current η and the inhibitory-to-inhibitory connectivity probability *P*_*II*_. The resulting two-dimensional bifurcation diagram is shown in Figure 1A.

The diagram reveals that the parameter space is divided into three qualitatively distinct dynamical regimes, demarcated by a Hopf bifurcation boundary. These regimes correspond to: (1) a stationary asynchronous firing state, (2) a low-frequency oscillatory state, and (3) a high-frequency oscillatory state. Although both (2) and (3) are both oscillatory states arising from a Hopf bifurcation, we categorize them as two distinct states due to differences in oscillation frequency and the neuronal population required to sustain the oscillation. For validation of the distinction between these two oscillatory states, see Supplementary Figure S1.

To validate the predictions of the macroscopic analysis, we simulated low-rank SNNs using parameter values sampled from each region. The corresponding raster plots are shown in Figures 1B–D. When both η and *P*_*II*_ are relatively low (green dot), the network exhibits asynchronous, non-oscillatory activity (Figure 1B). Increasing η while keeping *P*_*II*_ moderate (blue dot) drives the system into a high-frequency oscillatory regime, consistent with the onset of a supercritical Hopf bifurcation (Figure 1D). In contrast, increasing *P*_*II*_ while keeping η low (yellow dot) leads to low-frequency, intermittent population bursts (Figure 1C). These simulations confirm that the macroscopic bifurcation analysis accurately predicts the transitions between population-level dynamic states.

Although some quasi-oscillatory activity can be visually observed at the beginning and in the middle of the simulation in Figure 1B, we attribute this behavior to two factors: first, the simulation is not performed in an ideal infinite-size regime; second, the chosen parameters lie close to the Hopf bifurcation line. For simplicity, we did not further adjust other parameters to drive the network state sufficiently far from criticality. Nevertheless, power spectral analysis of longer simulations under this condition reveals the absence of prominent periodic components, distinguishing this state from genuine oscillatory regimes (see Supplementary Figure S4).

### 3.2 Go-Nogo task performance in the gamma oscillatory regime

**Figure 2:**
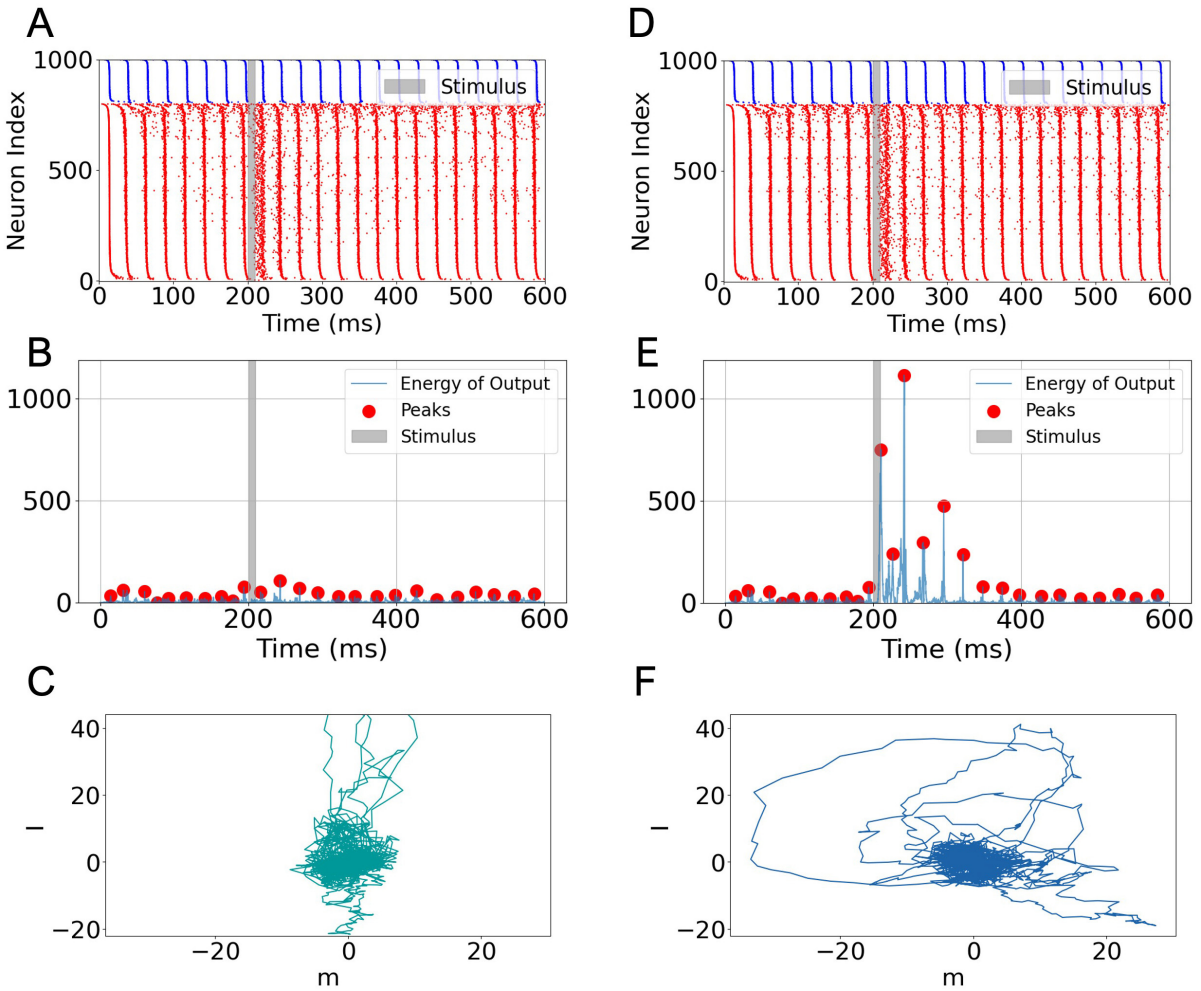
Task-related activity and output of a low-rank SNN under gamma oscillation. **(A)** Raster plot for a 600 ms trial under a “nogo” stimulus. Blue and red dots represent spikes from inhibitory and excitatory neurons, respectively. The gray-shaded region indicates stimulus onset and duration (10 ms). **(B)** Output energy (squared readout signal) corresponding to the “nogo” condition. Red dots mark energy peaks. **(C)** Projection of the postsynaptic current ${\text{I}}_{EE}^{\text{syn}}$ onto the ***m***-*I* plane under the “nogo” condition. **(D–F)** Same as **(A–C)** but for the “go” stimulus. A strong increase in output energy and broader projection trajectory are observed, indicating enhanced network response to task-relevant input.

Based on the bifurcation analysis results (Figure 1), we identified a parameter regime that induces gamma oscillation in the network. For the following task experiment, we selected (η = 2, *J*_*EE*_ = *J*_*EI*_ = *J*_*IE*_ = 0.1, *J*_*II*_ = 0.2), which reliably generates gamma-band activity. The oscillatory dynamics produced under these conditions are classified as ING-type (see Supplementary Figures S1A, B). During the task, task signal (**I**_*go*_ = ***n*** or **I**_*nogo*_ ⊥ ***n***, where $n_{i}\sim N\left(0,0.2\right),I_{nogo,i}\sim N\left(0,0.2\right)$) was applied for a duration of 10 ms, as indicated by the gray-shaded regions in Figures 2A, B, D, E.

Under gamma oscillation, the network successfully performed the Go-Nogo task (Figures 2B, E). Notably, when comparing the raster plots for the “nogo” (Figure 2A) and “go” (Figure 2D) stimuli, we observed that overall spike patterns remained similar, with only subtle increases in firing density during and after stimulation. This suggests that network-wide spiking activity does not substantially differentiate between stimuli at the level of raw raster plots.

However, when projecting the synaptic current ${\text{I}}_{EE}^{\text{syn}}$ onto the readout vector ***m*** and computing the output energy as the square of this projection, we observed a significant distinction: the output energy in response to the “go” signal exhibited a large transient increase, whereas no response to the “nogo” signal (compare Figure 2E vs. Figure 2B). A quantitative comparison of the peak output energy in response to Go and Nogo stimuli under the gamma oscillation state is presented in Section 4.1 of the Discussion.

This difference is further supported by visualizing the trajectory of synaptic current projections in the *m*-*I* plane. In the “go” condition, the trajectory exhibits larger excursions along the *m* direction (Figure 2F), while the “nogo” condition produces relatively confined movement near the origin (Figure 2C). These results are consistent with previous findings in low-rank rate networks (Mastrogiuseppe and Ostojic, 2018), and demonstrate that even when population spiking activity appears similar across conditions, meaningful stimulus selectivity can be extracted via structured readout directions.

### 3.3 Task performance modulated by the phase of gamma oscillation

**Figure 3:**
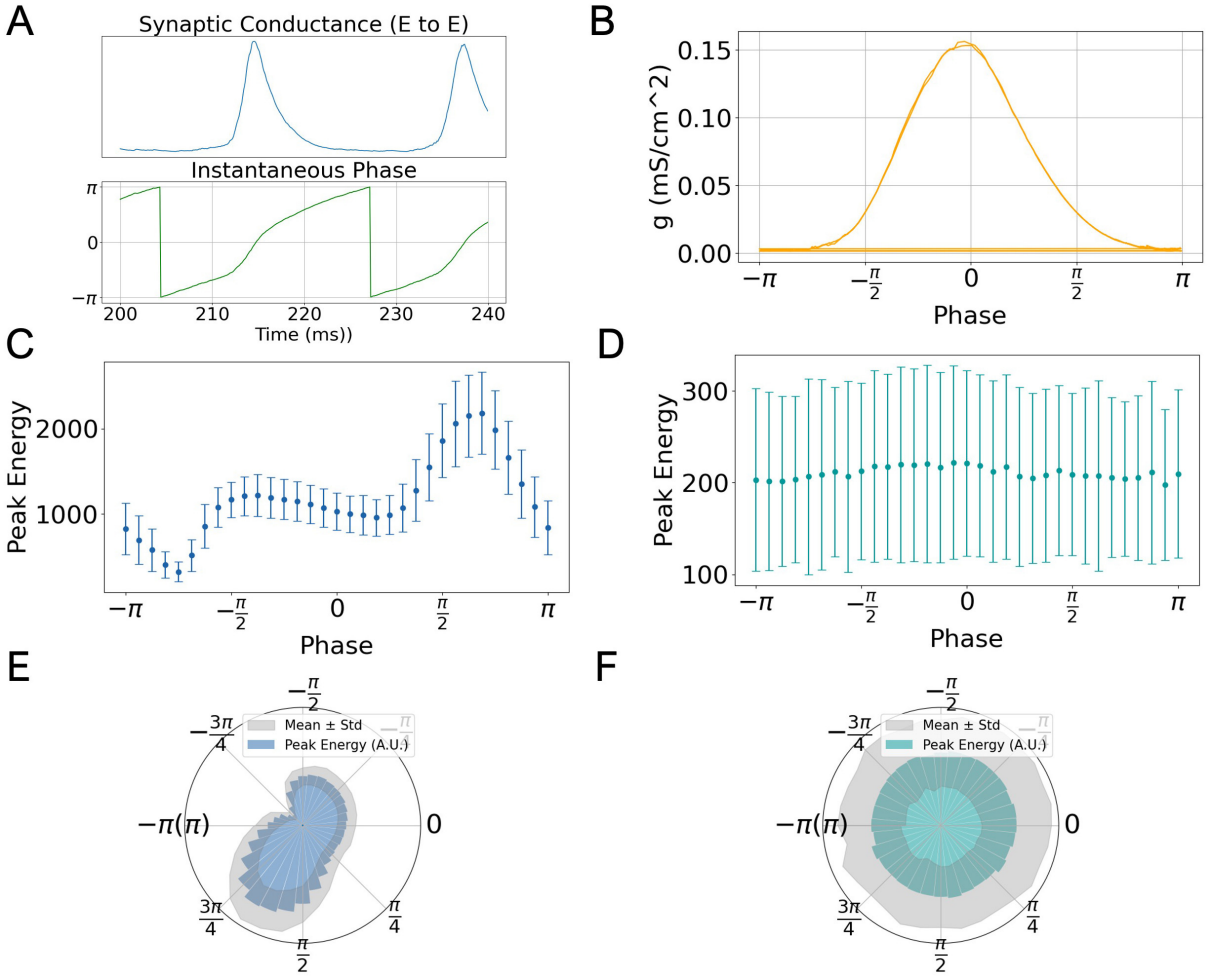
Phase-dependent modulation of task performance in low-rank SNNs. **(A)** Temporal evolution of excitatory-to-excitatory synaptic conductance *g*_*EE*_ (top) and its instantaneous phase extracted via Hilbert transform (bottom), during a representative oscillation window. **(B)** Average phase distribution of *g*_*EE*_, showing peak amplitude around phase 0. **(C)** Dependence of peak output energy ((μA/cm^2^)^2^) on stimulus phase for the go signal. Points represent means across 50 SNNs; error bars indicate standard deviation. **(D)** Same as **(C)** but for the nogo signal, showing no significant phase dependence. **(E, F)** Polar plots of data in **(C, D)**, respectively, highlighting phase-specific enhancement for the go stimulus and uniform response for the nogo stimulus.

To investigate how gamma oscillation modulate task performance in low-rank SNNs, we analyzed the network's response to stimuli applied at different phases of ongoing oscillatory activity. Since our spiking model does not explicitly define firing rate, we used the average excitatory-to-excitatory (E → E) synaptic conductance as a proxy for network activity. Specifically, the instantaneous phase of gamma oscillation was extracted by applying a Hilbert transform to the E → E synaptic conductance signal (Figure 3A, lower panel), which oscillates rhythmically over time (upper panel).

Figure 3B shows that the peak of synaptic conductance consistently occurs around phase 0, providing a reference point for aligning stimuli with different phases of the oscillatory cycle.

We divided each oscillatory cycle into 33 equally spaced phase bins and delivered 10 ms task-related input to the excitatory population at each phase point. We then quantified task performance as the peak output energy (squared readout) following the stimulus. This procedure was repeated across 50 independently generated low-rank connectivity matrices to account for variability.

Results show that in response to the preferred (go) stimulus, the peak output energy exhibits a clear dependence on the phase of gamma oscillation (Figure 3C). Notably, the response peaks just after the population burst, around phase π, and diminishes near the trough (phase −π). This phase-dependence is also clearly visible in polar coordinates (Figure 3E), where a directional bias in network responsiveness is observed.

In contrast, when presenting a non-preferred (nogo) stimulus, the network response shows no systematic variation across oscillation phases (Figures 3D, F). This suggests that phase-dependent modulation is specific to task-relevant inputs, consistent with the role of gamma oscillation in selective information routing.

As another evaluation, we investigated the phase dependence of the network's response speed. Specifically, we defined the reaction time of the task as the duration from stimulus onset to the time point at which the peak energy is reached. We examined how the reaction time in the Go-Nogo task varies with the phase of ongoing gamma oscillation. While the mean reaction time showed little dependence on the phase, the variability in reaction times exhibited a clear correlation with the peak output energy evoked at each phase. We found that the variability in reaction time was inversely proportional to the magnitude of the peak output energy (see Supplementary Figure S3). This finding suggests a potential link between reaction time and peak energy, which merits further investigation in future studies.

## 4 Discussion

### 4.1 Gamma oscillation enhances task-related performance

Here, by comparing the peak output energy under different states, we demonstrate that compared to the stationary firing state or low frequency oscillation state, the low-rank SNNs in gamma oscillation exhibits stronger response output energy.

**Figure 4:**
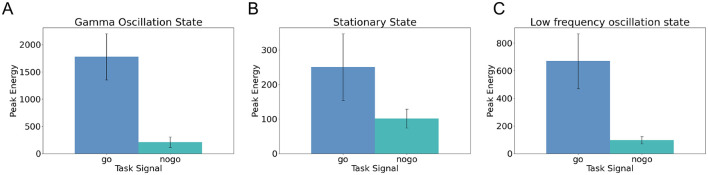
Comparison of low-rank SNN performance under different network states during the Go-Nogo task. **(A)** Peak output energy in response to Go and Nogo stimuli across 50 SNNs under the gamma oscillation state. **(B)** Peak output energy in response to Go and Nogo stimuli across 50 SNNs under the stationary firing state. **(C)** Peak output energy in response to Go and Nogo stimuli across 50 SNNs under the low-frequency oscillation state. Error bars indicate the standard deviation of the data.

Figures 4A–C show the peak output energy and standard deviations of 50 independently generated low-rank SNNs under three different network states: gamma oscillation, stationary firing, and low-frequency oscillation, during the Go-Nogo task. By comparing the peak responses across these three conditions, we found that the gamma oscillation state produced the highest peak output energy in response to Go stimuli, with average values ranging between 1,500 and 2,000 (μA/cm^2^)^2^, followed by the low-frequency oscillation state, and finally the stationary state. In contrast, the output energy in response to nogo stimuli remained low (between 100 and 200 (μA/cm^2^)^2^) across all three conditions. These results indicate that the gamma oscillation state selectively enhances responses to preferred stimuli.

Furthermore, by comparing the temporal profiles of output energy following Go stimulation under gamma oscillation and stationary firing states (Figure 2E vs. Supplementary Figure S2E), we observed that in the stationary state, the response to Go stimuli appears as a single transient energy peak that rapidly decays. In contrast, under gamma oscillation, the output energy exhibits multiple peaks lasting for approximately 150 ms, corresponding to about seven oscillation cycles. This finding reveals that gamma oscillation not only enhance peak output energy but also prolong the duration of network responses.

These findings suggest that gamma oscillation enable repeated activation and inhibition of task-relevant excitatory neurons, amplifying the network's response to relevant stimuli. This supports the hypothesis that oscillatory dynamics play an active role in enhancing cognitive processing, possibly by temporally organizing neural responsiveness.

PING (pyramidal-interneuron gamma) and ING (interneuron gamma) are two primary mechanisms underlying the generation of gamma oscillation (Buzsáki and Wang, 2012). Among them, ING can arise solely from inhibitory neurons without the involvement of excitatory neurons, whereas PING requires interactions between excitatory and inhibitory neurons. Distinguishing whether gamma oscillation are driven by ING or PING mechanisms is important for deepening our understanding of their functional diversity. In our model, although excitatory neurons are involved during gamma oscillation, we argue that the oscillation is primarily generated by recurrently coupled inhibitory neurons–characteristic of the ING mechanism. A key piece of evidence is that when the excitatory-to-inhibitory and inhibitory-to-excitatory connections are removed, inhibitory neurons alone are able to sustain gamma oscillation, which is a hallmark of the ING mechanism (see Supplementary Figures S1A, B). While our current focus is on ING-type oscillation, the involvement of excitatory neurons suggests that extending this framework to PING or even mixed PING/ING regimes represents an interesting direction for future research.

### 4.2 Conclusion and future perspectives

Neural oscillations, particularly in the gamma band, are widespread in the brain and play a fundamental role in cognition. *In vivo* studies have reported that the orientation selectivity of visual cortical neurons varies systematically with the phase of gamma oscillation (Womelsdorf et al., 2012; Vinck et al., 2010), suggesting that the phase of oscillatory activity modulates sensory processing. Inspired by this, we used low-rank SNNs based on the voltage-dependent theta neuron model to systematically investigate the effects of gamma oscillation on cognitive task performance.

Through bifurcation analysis of a macroscopic model, we characterized several distinct network states–from asynchronous stationary activity to robust gamma-band oscillation. We then applied the Go-Nogo task to evaluate performance across these regimes. Our results show that low-rank SNNs under gamma oscillation not only exhibit stronger responses to preferred stimuli but also display clear phase-dependent modulation of output energy. These findings align with experimental observations and suggest a functional role of oscillation phase in enhancing stimulus selectivity.

Importantly, while previous works have extended low-rank connectivity to spiking networks (Cimeša et al., 2023), they were typically limited to non-oscillatory regimes or did not rigorously enforce Dale's principle. In contrast, our model adheres to Dale's law and captures gamma-range population synchrony in biologically plausible excitatory-inhibitory (E-I) networks. This enables us to bridge structured connectivity, population-level oscillation, and cognitive function in a unified framework.

In particular, our simulations reproduce the experimentally observed phase-dependent responsiveness to stimuli (Figures 3C, E). Conceptually, this can be interpreted as follows: when a spontaneous population burst is imminent, external stimuli may be masked by ongoing activity, reducing network responsiveness. Conversely, following a burst–when neurons are more excitable and inhibition is reduced–external inputs are more likely to elicit a strong response. This explanation is supported by phase response function (PRF) analyses in similar models (Yoshikai et al., 2023). Moreover, such phase-dependent effects are not unique to gamma oscillation; for instance, alpha-phase has also been shown to modulate visual attention in humans (Mathewson et al., 2009).

In conclusion, our study provides a computational framework for interpreting how structured spiking networks encode and process information under oscillatory regimes. Future work could extend this framework to more complex tasks such as working memory or decision-making, to explore whether phase-dependent modulation generalizes across cognitive domains. For example, in the detection of noisy stimulus task introduced by Mastrogiuseppe and Ostojic (2018), the network should decide whether the noisy input has its average value exceeding a threshold. We can choose the vectors ***m*** and ***n*** to have a sufficiently large overlap. This configuration enables the low-rank network to integrate the stochastic stream, determine whether the running average exceeds a preset threshold, and generate the corresponding output. Because the task requires the network to retain accumulated information in the face of ongoing noise, it engages not only perceptual decision-making but also short-term working memory. A promising direction for future work is to test how delivering the stimulus at different phases of the ongoing oscillation influences success rates in this kind of evidence-integration task. Extending to higher rank would allow the network to exhibit richer dynamics, thereby enabling it to perform more complex tasks, such as multiple-choice tasks or decision-making tasks under different contents. Therefore, it is also a promising direction. Furthermore, our results may have practical implications for neurotechnology and brain-machine interfaces (BCIs). Designing stimulation protocols and decoding strategies that account for oscillation phase could enhance the efficacy and precision of neural control systems.

## Data Availability

The datasets presented in this study can be found in online repositories. The names of the repository/repositories and accession number(s) can be found in the article/Supplementary material.
