# Supplementary material for: Neural oscillation in low-rank SNNs: bridging network dynamics and cognitive function
Source: Front Comput Neurosci. 2025 Jun 4;19:1598138. doi: 10.3389/fncom.2025.1598138 (PMC12174079; doi:10.3389/fncom.2025.1598138)
Supplement: Supplementary file 1 [file Data_Sheet_1.pdf]

Supplementary Material

1 SUPPLEMENTARY FIGURES

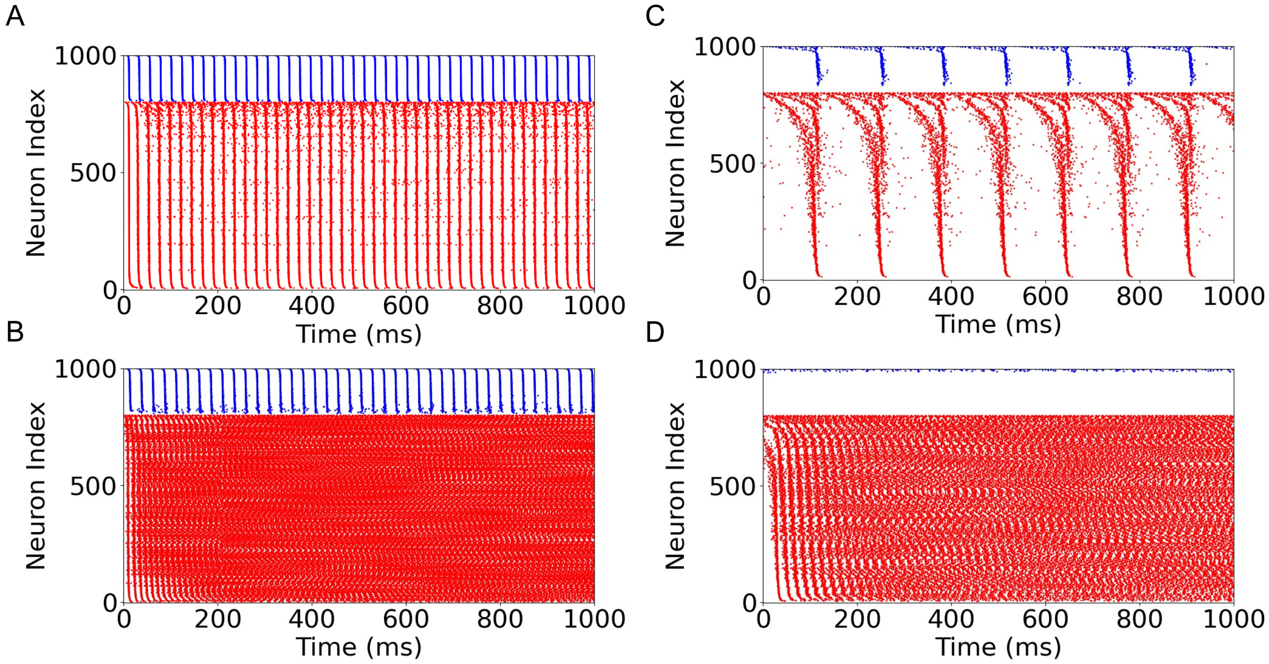

Figure S1. Assessment of oscillation type by removing excitatory-inhibitory (E–I) connectivity in a low-rank SNN. (A) Raster plot of a low-rank SNN under default parameter settings, showing synchronized gamma-band (40 Hz) oscillations in both excitatory and inhibitory populations. (B) Raster plot of the same network as (A) after manually removing all connections between excitatory and inhibitory populations. Excitatory neurons exhibit runaway firing due to the absence of inhibition, while inhibitory neurons continue to exhibit gamma oscillations despite the lack of excitatory drive. This result suggests that the observed gamma activity is dominated by inhibitory interneurons, consistent with ING (Interneuron Network Gamma) dynamics, in which inhibitory circuits alone can sustain oscillations independently of excitatory input. (C)Same as (A) except using parameter settings for low frequency oscillation. (D)Raster plot of the same network as (C) after manually removing all connections between excitatory and inhibitory populations. In contrast to (B), under the low-frequency oscillation state, the removal of EI and IE connections leads to a failure of inhibitory neurons to sustain oscillatory activity due to insufficient excitatory feedback. This indicates that such oscillations are dependent on the involvement of excitatory neurons and cannot arise from inhibitory dynamics alone. Based on this distinction, we treat this state as separate from the ING-supporting oscillatory state described in (A).

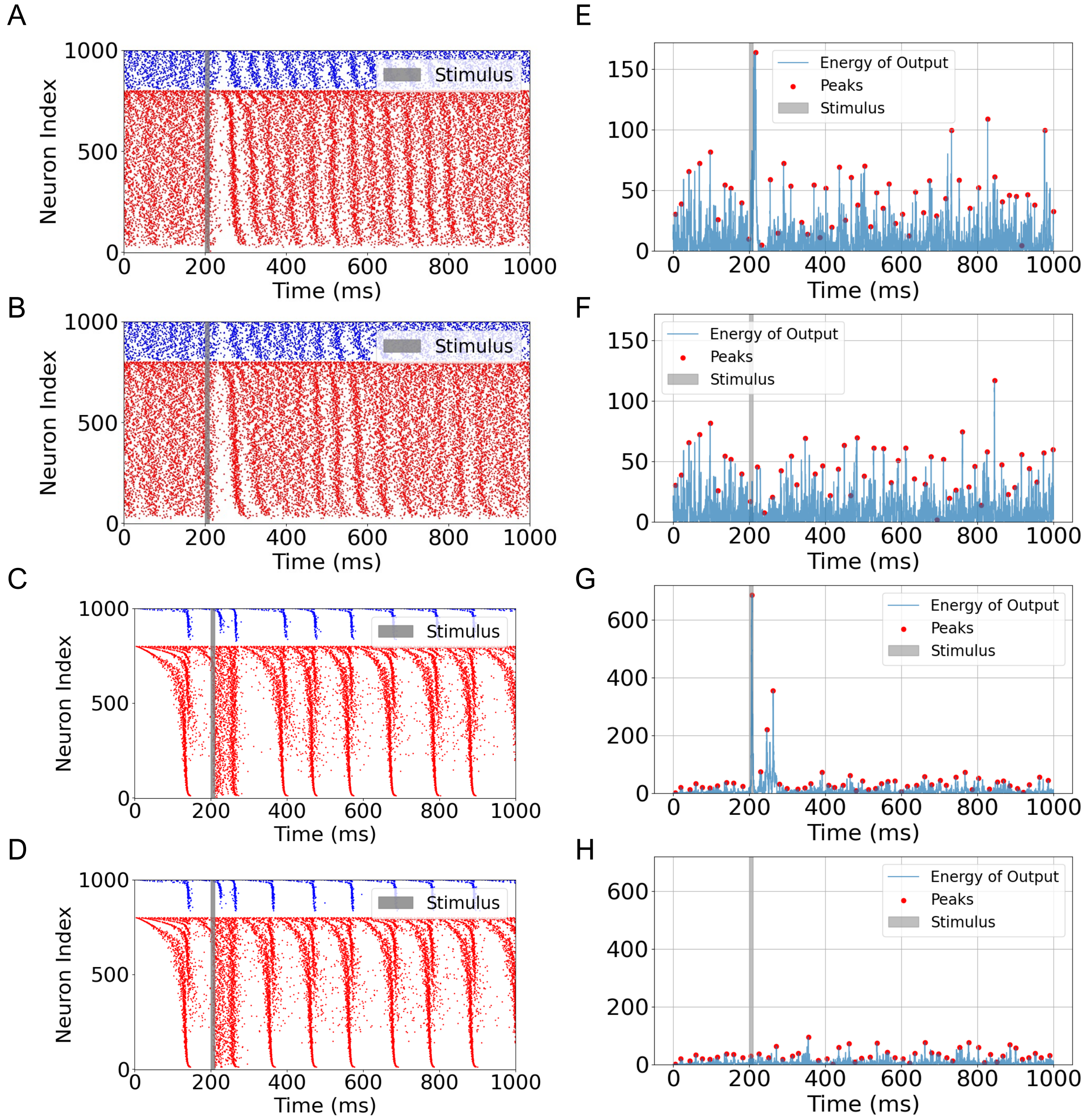

Figure S2. Validation of computational capability under stationary and low-frequency oscillatory states. To assess whether low-rank SNNs maintain stimulus selectivity across different population activity regimes, we tested the network's responses to Go and Nogo stimuli under stationary and low-frequency oscillatory conditions. (A, B) Raster plots of the network receiving Go (A) and Nogo (B) stimuli in the stationary firing state. (C, D) Raster plots of the network receiving Go (C) and Nogo (D) stimuli under low-frequency oscillations. (E–H) Corresponding peak output energy for each condition in panels (A–D), respectively. Results show that the network produces a clear increase in output energy in response to Go stimuli across both states, while Nogo stimuli elicit no significant output. These findings demonstrate that low-rank SNNs retain reliable stimulus discrimination performance regardless of the underlying network activity regime.

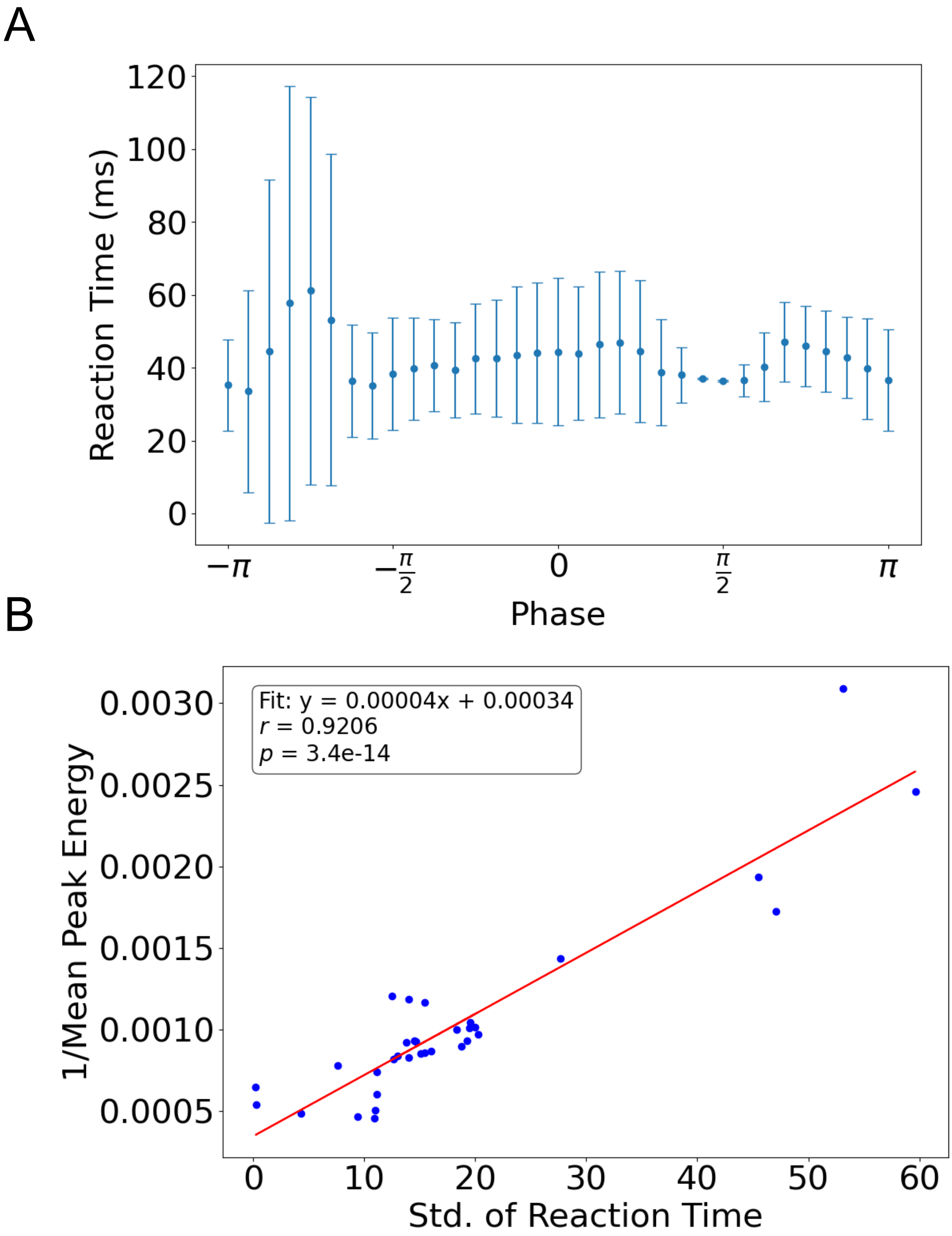

Figure S3. Quantitative analysis of reaction time in the Go-Nogo task under gamma oscillation (A) Dependence of reaction time on stimulus phase for the go signal. Points represent means across 50 SNNs; error bars indicate standard deviation. Reaction time is defined as the duration from stimulus onset to the moment when the network output reached its peak energy. The mean reaction time was approximately 40 ms. Notably, the variability of reaction time exhibited a clear dependence on the phase of oscillation. (B) Relationship between the standard deviation of reaction time and the inverse of the corresponding mean peak output energy. Blue dots represent the standard deviation of reaction time and the inverse of the mean peak energy for the same oscillation phase. The red line indicates the linear fit to these data points. Statistical analysis revealed a significant linear relationship between reaction time and the inverse of mean peak energy (Pearson correlation coefficient $r = 0.9206, p = 3.4 \times 10^{-14} \ll 0.05$). This indicates that the variability in reaction time is inversely proportional to the magnitude of peak energy.

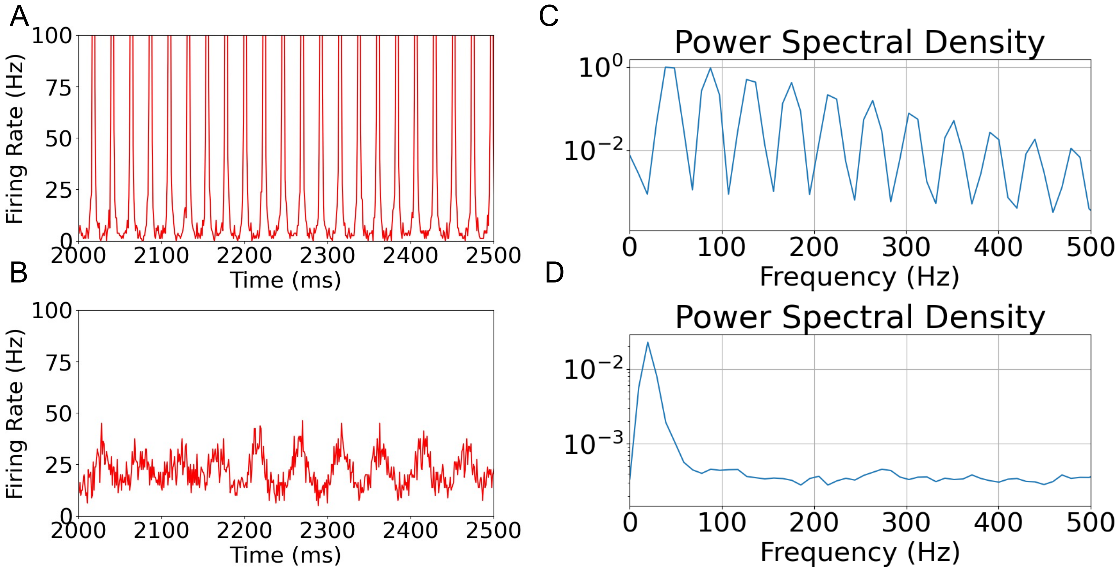

Figure S4. Analysis of simulation results under gamma oscillation and stationary state (A) Population firing rate of excitatory neurons calculated from a 5000 ms simulation under the gamma oscillation state using default parameters (10 ms time window for binning the raster plot). The figure displays the firing rate between 2000 and 2500 ms, showing clear, periodic sharp peaks at regular intervals, indicative of strong synchronized oscillations in the population. (B) Same as (A), but simulated under the stationary state parameters described in the main text. The population firing rate shows noisy fluctuations with occasional broad and blunt wave packets, lacking apparent periodicity. (C) Power spectral density (PSD) of the full 5000 ms population firing rate in (A). The gamma oscillatory signal exhibits a strong peak between 40 and 50 Hz, along with clear harmonics at integer multiples of the fundamental frequency, indicating that the underlying signal consists of periodic synchronized activity. (D) Same analysis as in (C) but for the stationary state. The PSD shows only a broad low-frequency component below 20 Hz and is nearly flat beyond 40 Hz, suggesting the absence of periodic activity and supporting the interpretation that no significant oscillation is present in this state.
